# Hydrogen‐Based Long‐Duration Energy Storage: Technologies, System Integration, and Techno‐Economic Performance

**DOI:** 10.1002/gch2.70139

**Published:** 2026-08-12

**Authors:** Reda A. Haggam, Syed Javed, Nguyen Van Minh, Rajkumar Sivanraju, Prajith Prabhakar

**Affiliations:** ^1^ Department of Chemistry Faculty of Science Islamic University of Madinah Madinah Saudi Arabia; ^2^ Department of Mechanical Engineering College of Engineering King Khalid University Abha Saudi Arabia; ^3^ Institute of Research and Development Duy Tan University Da Nang VietNam; ^4^ School of Engineering & Technology Duy Tan University Da Nang VietNam; ^5^ Department of Mechanical Engineering Faculty of Manufacturing Institute of Technology Hawassa University Hawassa Ethiopia; ^6^ Department of Mechanical Engineering Saveetha School of Engineering Saveetha Institute of Medical and Technical Sciences, SIMATS Chennai Tamil Nadu India

**Keywords:** digital twin optimization, hydrogen energy storage, long‐duration storage, power‐to‐hydrogen, techno‐economic analysis

## Abstract

The escalating integration of variable renewable energy sources has underscored the need for scalable, long‐duration energy storage (LDES) solutions to ensure power system reliability and resilience. Hydrogen (H_2_) based LDES is a rapidly emerging technology that offers substantial advantages in both retaining large volumes of energy for extended periods and facilitating integration across sectors (electricity, heat, transport). This review provides an extensive overview of H_2_‐based LDES technologies, focusing on the respective production routes, storage and reconversion methods, integration into overall systems, and optimization using digital tools. A systematic analysis of literature published between 2018 and 2026 is conducted to assess techno‐economic performance, life‐cycle environmental impacts, and reliability metrics, including loss‐of‐load expectation (LOLE) and effective load‐carrying capability (ELCC). The investigation underscores pivotal challenges related to efficiency losses, infrastructure preparedness, and operational complexity, while highlighting the significance of digital twins, artificial intelligence (AI), and market mechanisms in enhancing system performance. Prospective research avenues and policy implications are examined to support the large‐scale implementation of H_2_‐based energy systems in alignment with Sustainable Development Goal 7.

## Graphical abstract

Hydrogen‐based long‐duration energy storage (LDES) converts surplus renewable electricity into hydrogen through electrolysis, stores it using suitable storage technologies, and reconverts it into electricity, heat, or transport fuels. AI‐driven optimization, digital twins, and techno‐economic, environmental, and reliability assessments enable efficient, resilient, and low‐carbon energy systems that support large‐scale renewable integration and SDG 7.

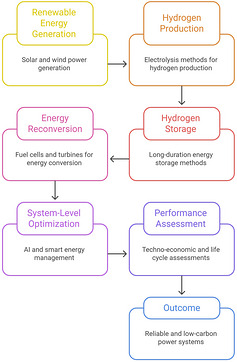

## 1 Introduction

The global energy industry is undergoing a historic transition as it addresses climate change, the shift away from fossil fuels, and long‐term energy security challenges. As renewable energy technologies (primarily solar and wind) are rapidly adopted in modern power systems, they are increasing low‐carbon electricity generation [1]. However, the variable and undispatchable nature of renewable energy technologies create major reliability, stability, and resource adequacy challenges for traditional power systems. Renewable energy is increasingly a share of the electric grid; however, as renewables grow in the overall grid mix, traditional power systems face greater difficulty maintaining a consistent balance between electricity generation and demand over longer periods, especially during periods of low renewable energy production [2, 3]. The problem is exacerbated by the seasonal mismatch between available energy (supply) and required energy (demand), which short‐duration energy storage technologies can't adequately manage [4].

Short‐duration energy storage systems, particularly those based on lithium‐ion batteries, have been extensively adopted for functions such as frequency regulation, voltage stabilization, and short‐term load balancing. Although these technologies have demonstrated efficacy for intra‐day operations, their limited storage duration, susceptibility to degradation under deep‐cycling conditions, and elevated costs at large scale limit their applicability for long‐term or seasonal energy storage solutions [5, 6]. Consequently, the transition toward high‐renewable energy systems has heightened the urgency for alternative solutions capable of providing energy storage over timescales ranging from days to months. In this regard, H_2_ has emerged as a highly versatile energy carrier, adept at overcoming the constraints of conventional storage technologies [7].

H_2_‐based energy systems enable the conversion of surplus renewable electricity into chemical energy via power‐to‐H_2_ processes, providing substantial energy storage and subsequent reconversion when needed. Unlike electrochemical storage technologies, H_2_ can be stored for extended periods without self‐discharge, transported over long distances, and used across sectors such as power generation, industry, transportation, and heating [8]. These characteristics render H_2_ particularly appealing for mitigating seasonal energy imbalances and fostering the large‐scale integration of renewable energy sources. Consequently, H_2_ has emerged as a pivotal component of national decarbonization strategies and long‐term energy planning frameworks globally [9].

In recent years, numerous nations have unveiled H_2_ roadmaps outlining ambitious objectives for green H_2_ production, infrastructure development, and industrial use. Large‐scale demonstration projects, H_2_ valleys, and power‐to‐X initiatives have further solidified H_2_’s position as an important element of future low‐carbon energy systems [10]. Correspondingly, the scholarly literature on H_2_ technologies has proliferated rapidly, encompassing a diverse array of topics, including electrolysis, storage methods, carrier systems, and end‐use applications. Despite this expanding corpus of work, most existing review studies adopt a fragmented perspective, focusing on individual components of the H_2_ value chain rather than analyzing H_2_ systems as cohesive energy infrastructures [11, 12].

A critical limitation of extant reviews is their restricted scope, which often segregates H_2_ production, storage, and utilization without accounting for interrelationships at the system level and operational constraints. Although numerous investigations provide comprehensive analyses of electrolysis technologies or H_2_ carriers, they seldom examine how these components function collectively within a power system [13]. Furthermore, the role of H_2_ as a medium for LDES is often articulated qualitatively, with scant attention to quantitative reliability metrics such as LOLE, expected energy not served (EENS), and ELCC. These metrics are pivotal for assessing the genuine contribution of H_2_ systems to grid adequacy and resilience; however, they are underrepresented in the prevailing literature [14, 15].

Another substantial gap pertains to the techno‐economic and environmental evaluation of H_2_‐based energy systems. Numerous studies report H_2_ production costs or life‐cycle emissions under inconsistent assumptions regarding electricity pricing, operational conditions, or system boundaries [16]. This lack of standardization hampers the ability to compare findings across studies and to draw robust conclusions about economic viability and environmental performance. Moreover, life‐cycle assessments (LCAs) frequently overlook emissions associated with infrastructure or the impacts of upstream electricity generation, leading to overly optimistic or incomplete evaluations of H_2_’s sustainability potential [17].

Concurrently, the accelerating digitalization of energy systems has opened new avenues for optimizing H_2_ production and utilization. Digital twins, AI, and advanced control algorithms are increasingly being employed to enhance electrolyzer operation, optimize storage dispatch, and improve system reliability [18]. These digital methodologies facilitate real‐time decision‐making, predictive maintenance, and adaptive control, thereby considerably augmenting the performance and economic feasibility of H_2_ energy systems. However, despite their growing relevance, digital optimization methodologies remain underexplored in the current body of H_2_‐focused review literature, particularly for LDES and grid‐scale deployment [19, 20].

In this context, there is a pressing need for a comprehensive, integrative review that assesses H_2_‐LDES at the system level. Such a review must transcend component‐level analysis and amalgamate technological performance, techno‐economic viability, environmental implications, reliability metrics, and digital optimization strategies within a cohesive framework [21]. Addressing these facets concurrently is essential to understanding the true potential of H_2_ as an important element of future low‐carbon energy systems and to identifying the critical barriers that must be overcome to facilitate large‐scale deployment [22].

This review aims to address these deficiencies by providing a structured, comprehensive assessment of H_2_‐LDES systems. In contrast to prior studies, this work synthesizes H_2_ production technologies, storage and carrier alternatives, reconversion pathways, and system integration strategies within a singular analytical framework. Emphasis is placed on evaluating H_2_’s contribution to power system reliability utilizing established adequacy metrics, while concurrently investigating techno‐economic performance and life‐cycle environmental impacts under realistic operational conditions. Additionally, the review underscores the emergent role of digital twins and AI in optimizing H_2_ energy systems and improving operational resilience.

By integrating production, storage, reconversion, TEA, LCA, reliability metrics, and digital optimization, this review provides a system‐level assessment of H_2_‐LDES. The review is intended to support researchers, system planners, and policymakers in comparing H_2_ storage pathways using consistent technical, economic, and environmental criteria. The analysis also identifies the conditions under which H_2_ storage is more suitable than short‐duration storage, particularly for multi‐day reliability, seasonal balancing, and sector‐coupling applications [22].

### 1.1 Definition and Classification of H_2_‐Based Long‐Duration Energy Storage

LDES refers to systems that can store energy for longer periods than conventional short‐duration storage. In power‐system studies, the lower boundary of LDES is commonly placed at approximately 8–10 h of discharge duration. In contrast, the upper boundary may extend to several days, weeks, or seasonal storage depending on the resource adequacy problem being addressed. This distinction is important because short‐duration storage mainly supports frequency regulation, ramping, and daily load shifting. In contrast, LDES is designed to manage longer periods of renewable underproduction, seasonal mismatch, and firm‐capacity needs in high‐renewable power systems [4].

In H_2_‐based systems, the level of LDES can be assessed using four practical criteria: discharge duration, stored energy capacity, storage loss over time, and the storage system's role in grid operation. Level 1 systems support extended intraday balancing over about 8–24 h. Level 2 systems provide multi‐day support during low‐wind or low‐solar periods. Level 3 systems address weekly to monthly energy deficits. Level 4 systems provide seasonal storage, where energy produced during high‐renewable periods is stored for later use during prolonged supply shortages. H_2_ is most relevant for Level 2 to Level 4 storage because molecular H_2_, chemical carriers, and geological storage can store large amounts of energy with low time‐dependent losses compared with electrochemical batteries [7].

A complete H_2_‐LDES system includes renewable or low‐carbon electricity input, H_2_ production, storage or conversion to a carrier, transport where required, and reconversion into electricity, heat, or mechanical energy. The suitability of a H_2_ pathway, therefore, cannot be assessed only from electrolyzer efficiency. It must also include storage duration, storage scale, compression or liquefaction energy, carrier‐conversion losses, reconversion efficiency, safety requirements, infrastructure readiness, and the value of reliability services provided to the power system [8].

For this review, H_2_‐LDES is defined as a H_2_‐based energy storage pathway that converts electricity or other low‐carbon energy into H_2_, stores it over intraday to seasonal timescales, and reconverts or uses it when energy demand, grid reliability, or sector‐coupling requirements justify the additional conversion losses. This definition distinguishes H_2_ storage for long‐duration grid support from its use solely as an industrial feedstock, transport fuel, or short‐term backup source [15]. The practical classification of H_2_‐LDES levels used in this review is summarized in Table 1.

**TABLE 1:** Classification of H_2_‐LDES levels according to discharge duration and storage function.

LDES level	Discharge duration	Main system function	Suitable H_2_ option	Main limitation	Refs.
Level 1: extended intraday	8–24 h	Daily renewable shifting and evening peak support	Compressed H_2_ with a proton exchange membrane fuel cell (PEMFC) or turbine reconversion	Lower round‐trip efficiency than batteries	[15]
Level 2: multi‐day	1–4 d	Support during low renewable generation periods	Compressed H_2_, liquid organic H_2_ carrier (LOHC), ammonia (NH_3_), or small cavern storage	Storage and reconversion cost	[22]
Level 3: weekly/monthly	4 d–1 month	Reliability support during extended renewable deficits	Salt cavern, depleted reservoir, ammonia, or LOHC	Site availability and infrastructure requirements	[56]
Level 4: seasonal	1–6 months	Seasonal energy balancing and strategic reserve	Geological storage, ammonia, or large‐scale carrier systems	High capital cost and regional dependence	[70]

Table 1 shows that H_2_‐LDES should be classified by discharge duration and system function rather than by storage medium alone. Compressed H_2_ is more suitable for extended intraday and multi‐day applications, whereas geological storage and H_2_ carriers are better suited to weekly, monthly, and seasonal storage. This classification separates grid‐scale long‐duration storage from short‐term backup and fuel‐only H_2_ applications by linking each level to discharge duration, system function, and suitable storage pathway.

## 2 Review Methodology

### 2.1 Literature Search Strategy

A complete systematic literature review was conducted to enable an extensive, replicable evaluation of H_2_‐LDES systems. The methodology for this review was developed in accordance with established principles of systematic reviews to reduce bias and enhance transparency in both article selection and analysis. Peer‐reviewed articles from the scientific databases (ScienceDirect, Scopus, Web of Science, IEEE Xplore) were systematically searched using keywords related to “energy systems,” “electrolysis technology,” and “power engineering.” Therefore, the temporal scope of this study was limited to publications from 2018 through 2026, thereby capturing recent developments in H_2_ energy systems technology, policy‐related developments in H_2_ energy systems, and changes in research trends regarding H_2_ energy systems.

A structured keyword strategy was systematically implemented by combining terms such as “H_2_ energy,” “long‐duration energy storage,” “power‐to‐H_2_,” “electrolysis,” “H_2_ storage,” “techno‐economic analysis,” “life cycle assessment,” and “grid reliability.” Boolean operators were carefully employed to refine search parameters and ensure comprehensive coverage of interdisciplinary studies spanning engineering, energy systems, and sustainability. Furthermore, forward and backward citation tracking was performed on highly cited articles to identify seminal studies that may not have been uncovered during the initial database search.

### 2.2 Screening and Selection Criteria

The acquired literature underwent a rigorous multi‐stage screening process to ascertain relevance and analytical rigor. In the initial stage, titles and abstracts were meticulously reviewed to exclude studies unrelated to H_2_‐based energy systems or long‐duration storage applications. The subsequent stage involved full‐text screening based on predefined inclusion and exclusion criteria. Studies eligible for inclusion in this analysis had to report either qualitative data and/or overall system performance metrics for H_2_ generation, H_2_ storage, H_2_ conversion back into a useful form, economic feasibility (techno‐economic), environmental impacts throughout its lifecycle, and/or how H_2_ can be integrated into electric grids. Conversely, studies that involved laboratory‐scale testing of individual materials, pure theory without quantifiable performance data, or non‐peer‐reviewed literature were systematically excluded.

As a result of this screening process, approximately 146 high‐quality peer‐reviewed journal articles were selected for in‐depth analysis. These selected studies collectively span a wide range of H_2_ technologies and applications, thereby ensuring a balanced representation of technical, economic, and system‐level perspectives.

### 2.3 Data Extraction and Analytical Framework

The selected studies were systematically categorized based on H_2_ production pathways, storage and carrier technologies, reconversion methodologies, and system integration strategies. Critical parameters such as energy efficiency, H_2_ production costs, capital and operational expenditures, carbon intensity, and operational lifespan were extracted wherever applicable. Reliability‐related metrics, including LOLE and capacity contribution, were also reported when available.

An integrated qualitative and quantitative synthesis approach was adopted for analysis. Qualitative assessments were employed to elucidate technological trends, identify research gaps, and address emerging challenges, while quantitative synthesis facilitated the comparison of performance metrics across diverse H_2_ pathways. Emphasis was placed on evaluating H_2_ systems from a power‐system perspective, encompassing their contributions to LDES, grid reliability, and sector coupling. This structured, methodological approach ensures a consistent, transparent, and system‐oriented evaluation of H_2_‐based energy systems and fosters meaningful comparisons across the existing literature.

**FIGURE 1:**
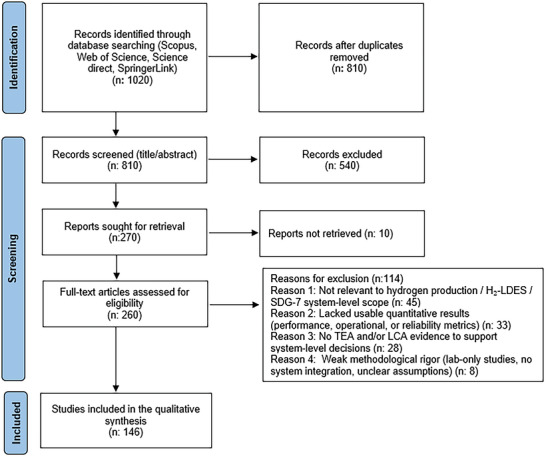
PRISMA flow diagram for literature selection and screening process.

Figure 1 illustrates the preferred reporting items for systematic reviews and meta‐analyses (PRISMA) flowchart, which encapsulates the systematic literature search and evaluation procedure. From 1020 entries recognized (Scopus, Web of Science, ScienceDirect, SpringerLink), 810 persisted post‐duplication elimination and were evaluated. Comprehensive texts were examined for 260 investigations (10 not obtained); 114 were dismissed based on predetermined criteria. A total of 146 studies were included in the qualitative synthesis.

## 3 Hydrogen Production Technologies for Long‐Duration Energy Storage

The production of H_2_ is a critical initial phase in establishing H_2_‐LDES systems, as it directly affects the system's overall efficiency, economic viability, environmental sustainability, and reliability. The appropriateness of H_2_ for extensive and prolonged applications depends not only on the production method but also on its operational adaptability, scalability, and compatibility with fluctuating renewable energy sources [23]. H_2_ production technologies can be broadly categorized into electrolysis‐based methods and thermochemical or hybrid methods. Each classification exhibits distinct technical attributes and systemic implications for LDES applications [24].

**FIGURE 2:**
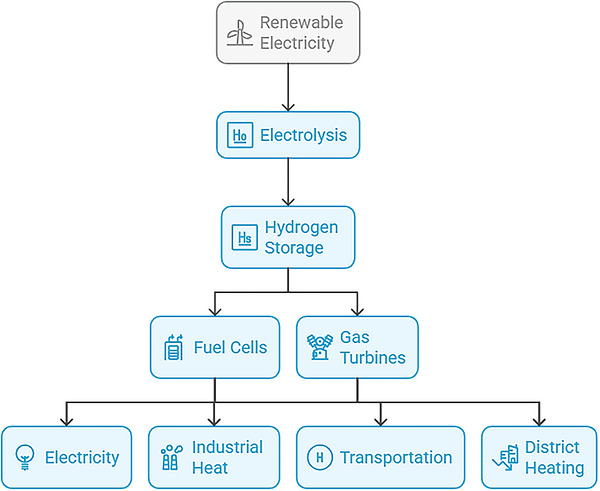
Architecture of a H_2_‐based long‐duration energy storage system for renewable energy integration.

Figure 2 depicts the Theoretical framework of a H_2_‐based LDES system, demonstrating the transformation of renewable electrical energy into H_2_, storage mechanisms, and reconversion via fuel cells and gas turbines to support electricity generation, industrial thermal energy, transportation, and district heating. The illustration underscores the significance of H_2_ in facilitating sector coupling, grid stabilization, and extended‐duration energy storage.

### 3.1 Electrolysis‐Based H_2_ Production

Electrolysis‐based H_2_ production is a central route for low‐carbon H_2_‐LDES when renewable or other low‐carbon sources supply the electricity input. Its relevance arises from the ability to convert variable electricity into a storable chemical energy carrier. However, the value of electrolysis for LDES depends on more than nominal conversion efficiency. It is influenced by electricity prices, electrolyzer capital costs, stack lifetime, operating flexibility, water requirements, capacity factor, degradation under dynamic loading, and the downstream costs of storage and reconversion [25].

Alkaline electrolysis (AEL), PEM electrolysis, anion exchange membrane (AEM) electrolysis, and solid oxide electrolysis show different levels of maturity and operational flexibility. Alkaline systems generally offer lower capital cost and long operating experience, but their slower dynamic response can limit direct coupling with highly variable wind and solar generation [26]. PEM systems provide faster response and higher current density, but they rely on expensive materials and require high‐purity water and careful membrane management. AEM systems aim to reduce catalyst cost while retaining operational flexibility, although durability remains a key barrier. Solid oxide electrolysis cell (SOEC) systems can reach high electrical efficiency when integrated with heat. Still, thermal cycling, materials degradation, and system complexity limit their near‐term use in highly dynamic LDES operation [27].

For LDES evaluation, electrolyzer comparison should include quantitative indicators such as electrical efficiency, operating temperature, technology readiness, expected lifetime, dynamic‐response capability, capital cost, and H_2_ production cost. Across the reviewed studies, electrolysis efficiency generally ranges from 60% to 90% [28]. At the same time, H_2_ production cost is strongly affected by the price of renewable electricity, electrolyzer utilization, stack replacement interval, and balance‐of‐plant costs [29]. These parameters determine whether H_2_ storage is suitable for multi‐day and seasonal applications rather than short‐duration services that can be supplied more efficiently by batteries [30].

Table 2 compares the major H_2_ production routes used in H_2_‐LDES by operating temperature, efficiency, technology readiness, advantages, limitations, and suitability. The suitability terms serve as comparative indicators of maturity, dynamic response, efficiency, cost, durability, and compatibility with variable renewable electricity. They should not be interpreted as universal rankings because the best technology depends on electricity price, operating profile, storage duration, and downstream reconversion requirements.

**TABLE 2:** Classification and quantitative comparison of H_2_ production technologies for long‐duration energy storage applications.

Technology	Operating temperature	Electrical efficiency (%)	Technology readiness level	Indicative cost or economic factor	Key advantages	Key limitations	Suitability for LDES	Refs.
AEL	60–90°C	60–70	9	Lower capital expenditure (CAPEX) than PEM; cost depends strongly on electricity price and utilization	Mature technology, long operating experience, and lower material cost	Slower dynamic response, lower current density, less suitable for frequent ramping	Moderate	[27]
PEM electrolysis	50–80°C	65–75	8–9	Higher CAPEX due to noble‐metal catalysts and membrane cost	Fast response, compact design, high current density, and good renewable coupling	High cost, iridium/platinum dependence, water purity requirement	High	[28]
AEM electrolysis	40–70°C	60–75	5–7	Potentially lower material cost if membrane durability improves	Non‐precious catalyst potential, flexible operation	Limited long‐term durability and membrane stability	Emerging	[29]
Solid oxide electrolysis	600–850°C	80–90	5–6	Cost depends on heat integration, stack durability, and thermal management.	High efficiency with heat integration, suitable for industrial sites	Thermal cycling, materials degradation, complex system operation	Large industrial‐scale use	[30]
Thermochemical routes, including Steam methane reforming (SMR) and gasification	700–1000°C	65–85	9	Economically mature, but low‐carbon use requires CCS and emission control	Large‐scale production, established industrial infrastructure	High carbon dioxide (CO_2_) emissions without CCS, methane leakage risk, feedstock dependence	Limited by carbon capture and storage (CCS)	[31]

The quantitative comparison in Table 2 indicates that electrolysis‐based production is most suitable for low‐carbon H_2_‐LDES when low‐cost renewable electricity and sufficient operating hours are available. AEL offers lower cost and maturity but has limited flexibility under variable renewable input. PEM electrolysis is better suited to dynamic operation but incurs higher capital costs due to the need for membranes and noble‐metal catalysts. SOEC provides higher efficiency when heat is available, but its use in LDES is constrained by thermal cycling and durability. Therefore, electrolyzer selection should be linked to storage duration, expected capacity factor, electricity price profile, and reconversion pathway rather than to efficiency alone.

#### 3.1.1 Alkaline Electrolysis

AEL represents the most developed and commercially prevalent electrolysis technology, having been employed in industrial contexts for several decades. It operates with a liquid alkaline electrolyte, most commonly potassium hydroxide, and is characterized by relatively low CAPEX and extended system lifetimes. AEL systems are particularly well‐suited to large‐scale H_2_ production under consistent operating conditions and are frequently used in industrial H_2_ generation [32].

Nonetheless, alkaline electrolyzers exhibit limited dynamic responsiveness and reduced efficiency under variable power inputs, thereby limiting their ability to match the profiles of variable renewable energy sources closely [33]. This constraint undermines their effectiveness in scenarios that require rapid ramping or frequent load changes, which are common in energy systems dominated by renewables. Despite these limitations, their durability and low cost continue to render them appealing for baseload H_2_ production in long‐duration storage contexts [34].

#### 3.1.2 Proton Exchange Membrane (PEM) Electrolysis

PEM electrolysis has attracted considerable scholarly attention due to its high current density, compact architecture, and exceptional dynamic responsiveness. PEM electrolyzers can rapidly modulate output in response to fluctuations in renewable energy supply, making them particularly well‐suited for integration with solar and wind energy. Their proficiency in operating at partial load and accommodating frequent start‐stop cycles confers a significant advantage for LDES applications [35].

However, despite these advantages, PEM electrolyzers depend on noble‐metal catalysts, such as platinum and iridium, which increase capital costs and pose challenges for supply chain sustainability. Current research efforts are focused on reducing reliance on precious‐metal catalysts, enhancing membrane durability, and prolonging system lifetimes to support commercial viability [36].

#### 3.1.3 Anion Exchange Membrane Electrolysis

AEM electrolysis has emerged as a compelling alternative that combines the benefits of alkaline and PEM technologies. AEM systems are designed to operate with non‐precious metal catalysts, ensuring high efficiency and operational flexibility. This methodology can reduce system expenditures and enhance scalability [29]. Although AEM electrolysis is currently in a relatively nascent stage of development, recent investigations suggest rapid improvements in membrane stability, ionic conductivity, and operational longevity. Should these obstacles be effectively surmounted, AEM electrolyzers could assume a pivotal role in forthcoming H_2_‐based energy systems [37].

#### 3.1.4 Solid Oxide Electrolysis

SOEC operates at elevated temperatures and achieves the highest electrical efficiency among electrolysis methods, particularly when integrated with industrial waste heat or high‐temperature renewable energy sources. The elevated operating temperature reduces electrical energy requirements and enables more efficient H_2_ generation [38].

Nonetheless, SOEC systems face challenges related to material degradation, thermal cycling, and system complexity. Prolonged stability under dynamic operational conditions constitutes a critical impediment to large‐scale implementation. However, SOEC technology demonstrates significant promise for industrial H_2_ generation and for integrated energy systems where high‐temperature heat is readily available [39]. Across all electrolysis technologies, system efficiencies generally range from 60% to 85%, while H_2_ production costs range from 2 to 6 USD per kilogram, contingent on electricity pricing, capacity factor, and system longevity. For LDES, the flexibility, durability, and scalability of electrolyzers are pivotal determinants of overall system efficacy [40].

From a system perspective, alkaline and PEM electrolysis are the most relevant near‐term options for H_2_‐LDES, but they serve different operating cases. Alkaline systems are better suited to high‐utilization operation where electricity supply is relatively stable. In contrast, PEM systems are more suitable for applications requiring rapid ramping and frequent start‐stop operation. AEM systems may reduce material cost if membrane lifetime and stability improve, whereas SOEC systems are better matched to industrial sites with available high‐temperature heat. Therefore, the choice of electrolyzer should be linked to the expected storage duration, renewable profile, capacity factor, and downstream storage medium, rather than being selected solely by stack efficiency [40].

### 3.2 Thermochemical and Hybrid H_2_ Production Pathways

Thermochemical and hybrid H_2_ production pathways remain relevant to H_2_‐LDES because they can use existing industrial assets, biomass resources, waste streams, or heat integration opportunities. These pathways include SMR, coal or biomass gasification, methane pyrolysis, waste‐derived H_2_ production, and hybrid systems that combine renewable electricity with thermal or chemical conversion routes. Their suitability depends on carbon intensity, feedstock availability, process efficiency, carbon‐capture performance, water use, residue handling, and compatibility with long‐duration storage infrastructure [41].

SMR is currently the dominant industrial H_2_ production route because of its maturity and large‐scale operating experience. However, its role in low‐carbon LDES is limited unless CCS is included and upstream methane leakage is controlled. Without carbon capture, fossil‐based H_2_ can exceed the life‐cycle emissions of renewable H_2_ produced via electrolysis. With carbon capture, emissions can be reduced, but residual CO_2_, methane leakage, capture‐rate uncertainty, and additional capital cost remain important limitations. For this reason, fossil‐derived H_2_ should be treated as a transitional or region‐specific pathway rather than a universal LDES solution [42].

#### 3.2.1 Conventional Thermochemical Pathways

SMR remains the dominant industrial H_2_ production route because of its maturity, established supply chains, and large‐scale operating experience. However, its suitability for low‐carbon H_2_‐LDES depends on the integration of carbon capture, control of methane leakage, and the availability of CO_2_ transport and storage infrastructure. Without carbon capture, fossil‐derived H_2_ has high life‐cycle emissions and is not suitable for low‐carbon storage pathways. With carbon capture, SMR may support transitional or region‐specific deployment, but residual emissions, capture‐rate uncertainty, and infrastructure cost must be included in system assessment [42].

Additionally, because the process generates H_2_ from a fossil fuel source, SMR also generates large quantities of CO_2_ unless carbon capture and sequestration technologies are employed. In addition to SMR, coal and biomass gasification processes may produce H_2_‐rich syngas; however, the emissions and by‐product handling associated with these processes must be carefully managed [43]. While these methodologies are not inherently low‐carbon, integrating them with carbon capture systems can substantially mitigate emissions, facilitating a transition to cleaner H_2_ production. Nevertheless, costs, infrastructure requirements, and public acceptance continue to pose significant challenges [44].

#### 3.2.2 Biomass‐Based and Waste‐Derived Pathways

Biomass gasification and pyrolysis offer renewable alternatives for H_2_ production by utilizing agricultural residues, forestry waste, and organic by‐products. These pathways offer the potential for negative or near‐zero carbon emissions when sustainably sourced feedstocks are employed. However, challenges such as feedstock variability, tar formation, and complex process control limit large‐scale implementation [45]. Despite these hurdles, biomass‐based H_2_ production plays a vital role in circular economy frameworks and can synergistically complement electrolysis‐based systems, particularly in regions with abundant biomass resources [46].

#### 3.2.3 Hybrid H_2_ Production Systems

Hybrid H_2_ production systems combine electrochemical and thermochemical processes to enhance overall efficiency and system adaptability. Illustrative examples include integrating renewable electricity with biomass gasification or using waste heat from industrial processes to drive electrolysis. These hybrid configurations facilitate greater energy utilization and lower carbon intensity whilst concurrently improving resilience through diversified energy sources [47]. From the perspective of LDES, hybrid systems provide a complementary alternative to electrolysis‐based methods, particularly in energy systems characterized by seasonal resource variability or constrained availability of renewable electricity [48].

Hybrid production systems can improve resilience where a single energy resource is insufficient across seasons. For example, biomass gasification may complement renewable electrolysis when wind or solar output is low, while waste heat can reduce the electrical input required for high‐temperature electrolysis. The main limitations are process control, feedstock variability, tar or impurity management, system complexity, and the need for consistent carbon accounting. In LDES planning, hybrid pathways are most useful when they reduce seasonal supply risk without increasing life‐cycle emissions or storage‐chain complexity [40, 48].

For thermochemical and hybrid routes, quantitative evaluation should include production efficiency, feedstock cost, carbon intensity, carbon capture rate, CO_2_ transport requirements, by‐product handling, and compatibility with long‐duration storage. Biomass and waste‐derived pathways can reduce fossil dependence, but their deployment is limited by feedstock variability, tar formation, ash handling, logistics, and process control [48, 49].

## 4 Hydrogen Storage and Carrier Technologies

H_2_ storage for LDES can be grouped into physical storage, chemical carrier storage, and geological storage. Physical storage retains molecular H_2_ as compressed H_2_, liquid H_2_, or cryo‐compressed H_2_. Chemical carriers store H_2_ in chemically bound forms such as ammonia, methanol, formic acid, and LOHCs. Geological storage uses salt caverns, depleted gas reservoirs, or aquifers to store large quantities of H_2_ over long durations. The suitability of each option depends on discharge duration, storage scale, volumetric density, conversion losses, cost, safety, maturity, regional availability, and compatibility with production and reconversion systems [50].

Table 3 compares H_2_ storage and carrier options based on energy density, storage conditions, scalability, and suitability for LDES. The suitability terms are based on storage duration, scalability, energy penalty, infrastructure requirements, technology maturity, and regional availability. They are therefore application‐specific classifications rather than universal consensus categories.

**TABLE 3:** Characteristics of H_2_ storage and carrier technologies for long‐duration energy storage.

Storage method	Gravimetric energy density (kWh kg^−1^)	Volumetric energy density (kWh m^−^^3^)	Storage conditions	Scalability	Suitability for LDES	Refs.
Compressed H_2_	33.3	1.2–2.0	350–700 bar	Moderate	Limited	[51]
Liquid H_2_	33.3	2.3–2.5	−253°C	High	Moderate	[52]
Cryo‐compressed H_2_	33.3	2.5–3.0	Cryogenic + pressure	Medium	Emerging	[53]
LOHC	1.9–2.2	1.9–2.3	Ambient	High	High	[54]
Ammonia	5.2	3.0–3.5	−33°C or pressurized	Very high	Very high	[55]
Geological Storage	—	Very high	Underground caverns/reservoirs	Very high	Very high	[56]

Table 3 classifies storage suitability using application‐specific criteria, including storage duration, scalable energy capacity, energy penalty, infrastructure requirement, technology maturity, and regional availability. In this review, suitability for LDES is assessed using six criteria: achievable storage duration, scalable energy capacity, energy penalty, infrastructure requirement, technology maturity, and regional availability. Compressed H_2_ is classified as limited for seasonal LDES due to its low volumetric density and high tank costs at large scale. Liquid H_2_ is classified as moderate because it improves density but requires energy‐intensive liquefaction and boil‐off management. LOHC and ammonia are more suitable for transportable, long‐duration storage because they can use liquid fuel or chemical logistics infrastructure. Geological storage is the strongest option for large, stationary, and seasonal storage when suitable formations are available.

### 4.1 Physical H_2_ Storage Technologies

Physical storage involves retaining H_2_ in its molecular form under elevated pressure or low temperature. These methodologies are technologically advanced and widely used in industrial and mobility contexts, although their suitability for large‐scale energy storage varies considerably [57].

#### 4.1.1 Compressed H_2_ Storage

Compressed H_2_ storage is the most established physical storage method for H_2_. It stores H_2_ gas at elevated pressures, typically 350–700 bar, in high‐pressure vessels or storage systems. Its advantages include fast charging and discharging, mature safety protocols, and suitability for decentralized applications such as refueling stations, backup power, and short‐ to medium‐duration storage. These characteristics make compressed H_2_ useful for distributed energy systems and mobility‐related infrastructure [58].

The main limitation of compressed H_2_ is its low volumetric energy density compared with liquid H_2_ (LH_2_), ammonia, and geological storage. Compression also consumes a portion of the stored energy, typically reported as 7%–15%, depending on the pressure level, compressor design, and system boundary [55]. High‐pressure tanks increase capital cost, require strict safety management, and occupy considerable space when scaled to grid‐level or seasonal storage. Therefore, compressed H_2_ is better suited to short‐ and medium‐duration decentralized applications than to large seasonal storage, unless it is combined with underground or large stationary storage infrastructure [59].

#### 4.1.2 Liquid H_2_ Storage

Liquid H_2_ storage offers a higher volumetric energy density than compressed H_2_ and is useful for compact storage or long‐distance transport. H_2_ liquefaction occurs at approximately −253°C, enabling higher storage density but requiring cryogenic infrastructure, insulation, boil‐off control, and specialized handling systems. These requirements make liquid H_2_ more suitable for transport‐ and export‐oriented supply chains than for low‐cost, stationary, seasonal storage [60].

Despite these benefits, the liquefaction process is highly energy‐intensive, consuming approximately 25%–35% of the H_2_’s lower heating value. Additionally, boil‐off losses during storage and transportation further reduce the system's overall efficiency. The need for cryogenic insulation and specialized handling infrastructure further amplifies capital and operational complexities [61]. Consequently, LH_2_ storage is well‐suited to applications in which high energy density and long‐distance transport are prioritized over efficiency considerations, such as maritime transport and export‐oriented H_2_ supply chains [62].

#### 4.1.3 Cryo‐Compressed H_2_ Storage

Cryo‐compressed H_2_ storage combines low‐temperature storage with moderate‐to‐high pressure to increase volumetric density compared with conventional compressed H_2_ while reducing some boil‐off limitations associated with LH_2_ storage [63]. Although cryo‐compressed systems are still under development, they have potential for applications that require higher storage density and greater operational adaptability. However, the high complexity of these systems and the current lack of extensive commercial experience substantially inhibit large‐scale implementation [64].

### 4.2 Chemical H_2_ Storage and Carrier Systems

Chemical H_2_ storage systems retain H_2_ in chemically bound forms and can improve transportability compared with compressed H_2_ or liquid H_2_ (LH_2_). LOHCs, ammonia, methanol, and formic acid are relevant where long‐distance transport, import dependence, or storage at ambient conditions is required. However, these carriers require conversion and reconversion steps, which add energy losses, catalyst requirements, safety considerations, and additional infrastructure needs [65].

#### 4.2.1 Liquid Organic H_2_ Carriers

LOHCs employ reversible H_2_ation and deH_2_ation reactions to sequester H_2_. The advantages of LOHCs are multifaceted, including ambient‐temperature storage, compatibility with existing liquid‐fuel infrastructure, and enhanced safety due to their low flammability. These attributes make LOHCs particularly appealing for transporting H_2_ over long distances and for seasonal storage [66].

Nonetheless, LOHC systems incur energy penalties due to H_2_ation and deH_2_ation, thereby diminishing overall system efficiency. Challenges remain in catalyst longevity, reaction kinetics, and thermal management. Despite these obstacles, LOHCs are progressively being evaluated for extensive H_2_ logistics, particularly where safety and compatibility with existing infrastructure are prioritized [67].

#### 4.2.2 Ammonia as a H_2_ Carrier

Ammonia has emerged as a highly promising H_2_ carrier, primarily due to its substantial H_2_ content, well‐established global infrastructure, and the relative ease of liquefaction under moderate conditions. Ammonia can be stored and transported via existing fertilizer distribution networks, making it particularly suitable for international H_2_ trade and seasonal energy storage applications [68].

H_2_ can be extracted from ammonia via catalytic cracking or utilized directly as a fuel in combustion or fuel cell systems. While ammonia offers high energy density and cost‐effective storage, challenges remain regarding conversion efficiency, nitrogen oxide emissions, and toxicity. Ammonia is therefore relevant for international H_2_ transport and seasonal storage, but its use requires careful management of toxicity, cracking efficiency, nitrogen oxide emissions, and safety standards [69].

### 4.3 Geological H_2_ Storage

Salt dome caverns, depleted gas reservoirs, and aquifers provide suitable geological storage options for H_2_ at scales ranging from gigawatt‐hours to terawatt‐hours. The three geological options provide a means to store H_2_ in sufficient quantities to support both seasonal H_2_ storage and grid stabilization [70].

Salt dome caverns offer an attractive option for H_2_ storage due to their low permeability and proven track record in natural gas storage. Because they can handle high injection and withdrawal rates, they also provide the flexibility to support both short‐term balancing and longer‐term H_2_ storage needs. Although depleted hydrocarbon reservoirs offer significant storage capacity, they must undergo a thorough assessment before use to identify potential chemical interactions, leakage risks, and microbial activity [71].

Geological H_2_ storage is essential for enabling H_2_‐LDES at both national and regional scales. However, site‐specific geological evaluations, regulatory frameworks, and public acceptance are critical considerations for the broad implementation of such technologies [72].

### 4.4 Regional Suitability of H_2_ Storage Options

H_2_ storage suitability varies strongly by region because geological formations, renewable resources, industrial demand, port infrastructure, and pipeline availability are unevenly distributed. Regions with salt caverns or suitable depleted gas reservoirs have better prospects for stationary seasonal storage. In contrast, countries with limited geological storage may rely more on ammonia, LH_2_, or LOHC‐based import chains. This distinction is important because a storage option that is technically suitable in one region may be costly or impractical in another [70].

Salt caverns are well suited to large‐scale storage because they can provide high deliverability, low leakage risk, and repeated injection–withdrawal operations. Their use is more practical in regions where salt formations are near renewable generation, H_2_ hubs, or industrial demand centers. Depleted gas reservoirs and aquifers may offer greater theoretical storage capacity. Still, they require site‐specific assessment of caprock integrity, cushion gas demand, microbial activity, geochemical reactions, and H_2_‐loss mechanisms. These factors make geological storage a regional resource rather than a uniformly available solution [71].

For countries with limited domestic storage geology but strong H_2_ demand, chemical carriers provide a more practical storage and transport pathway. Ammonia benefits from an established global infrastructure for handling and shipping, but it incurs conversion losses, toxicity risks, and NOx emissions during direct use. LOHC systems can leverage liquid‐fuel logistics and operate under near‐ambient conditions, but the heat demand for deH_2_ation and catalyst durability affect overall efficiency. LH_2_ is relevant where high transport density is needed, although liquefaction energy and boil‐off losses limit its role in low‐cost stationary LDES [72].

Table 4 shows that H_2_ storage selection is strongly region‐specific and depends on local geology, infrastructure, and end‐use demand. Regions with salt caverns or depleted gas reservoirs are better suited for large‐scale geological storage, while import‐dependent systems may rely on ammonia, LH_2_, or LOHC supply chains. Industrial clusters and decentralized systems require more flexible options such as compressed H_2_, pipelines, or ammonia, but these routes face limitations related to cost, safety, and volumetric energy density.

**TABLE 4:** Regional suitability of H_2_ storage options.

Region or system type	Natural/storage endowment	Suitable H_2_ storage route	Main advantage	Main limitation	Refs.
Regions with salt caverns	Salt formations and gas‐storage experience	Geological storage	High‐capacity seasonal storage	Site‐specific availability	[70]
Regions with depleted gas reservoirs	Existing subsurface infrastructure	Geological storage after assessment	Large potential capacity	Leakage, cushion gas, and geochemical risk	[71]
Import‐dependent countries	Ports and chemical import infrastructure	Ammonia, LH_2_, LOHC	Suitable for long‐distance supply chains	Conversion losses and safety requirements	[68]
Industrial clusters	Concentrated H_2_ demand	Compressed H_2_, pipelines, ammonia	Shared infrastructure and demand aggregation	High initial infrastructure cost	[73]
Decentralized systems	Small‐scale demand and limited pipelines	Compressed H_2_	Fast response and modularity	Low volumetric density	[59]

The regional comparison shows that H_2_ storage planning should not rely on a single preferred pathway. Geological storage is more suitable where subsurface conditions are favorable, while ammonia, LH_2_, and LOHC routes are more relevant for import‐dependent or port‐linked energy systems.

### 4.5 Implications for Long‐Duration Energy Storage Systems

The choice of H_2_ storage technology substantially impacts the performance metrics, economic viability, and operational practicality of LDES systems. Physical storage methods are particularly advantageous for short‐term, decentralized applications, whereas chemical carriers and geological storage mechanisms facilitate large‐scale, seasonal energy balancing. In practical applications, a combination of various storage technologies is likely necessary to meet the heterogeneous operational demands of energy systems [73].

From a systemic perspective, H_2_ storage technologies should be assessed not solely on parameters such as energy density and efficiency, but also on criteria including scalability, safety, compatibility with existing infrastructure, and integration with production and conversion technologies. Future investigative efforts should prioritize reducing storage costs, improving round‐trip efficiency, and developing integrated storage strategies that underpin the reliability of low‐carbon energy systems [74].

## 5 Hydrogen Utilization and Energy Reconversion

The processes of H_2_ utilization and reconversion constitute the terminal phase of H_2_‐LDES systems. They are instrumental in shaping their overall efficacy, operational adaptability, and economic feasibility. After H_2_ is produced and stored, it must be converted back into usable forms of energy, such as electricity, thermal energy, or mechanical power [75]. The selection of a suitable H_2_ conversion technology depends on the application conditions, as well as response time, efficiency, scalability, and compatibility with existing energy systems. Conversion to H_2_ can occur via several methods (electrochemical, thermochemical, and combustion), each with different advantages and disadvantages for long‐term energy storage [76].

### 5.1 Fuel Cell‐Based Energy Reconversion

Fuel cells convert H_2_ into electricity through electrochemical reactions and are important reconversion devices in H_2_‐LDES systems. PEMFCs and solid oxide fuel cells (SOFCs) are the most relevant types for storage and power applications. PEMFCs operate at relatively low temperatures, start rapidly, and provide good load‐following capability. These characteristics make them suitable for grid balancing, backup power, microgrids, electric buses, heavy‐duty trucks, material‐handling vehicles, and other applications that require dynamic operation. In H_2_‐LDES systems, PEMFCs can provide dispatchable electricity during renewable supply shortages, although their value depends on stack lifetime, H_2_ purity, system cost, and operating profile [77].

The main technical limitations of PEMFCs are catalyst cost, membrane degradation, water and thermal management, sensitivity to H_2_ impurities, and the need for balance‐of‐plant components such as compressors, humidifiers, cooling systems, and power electronics. These factors increase system cost and maintenance requirements. For transport applications, fuel cells are better suited to long‐range or high‐utilization vehicles than to short‐range passenger vehicles, where batteries are often more efficient. In heavy‐duty electric mobility, fuel‐cell systems can reduce refueling time and increase driving range. Still, their deployment depends on a reliable H_2_ supply, refueling infrastructure, and lower delivered H_2_ costs [78].

Fuel‐cell cost remains a major constraint for transport and stationary reconversion. The cost of a PEMFC system is affected by platinum‐group‐metal loading, membrane durability, stack power density, manufacturing scale, and balance‐of‐plant complexity. For electric buses, heavy‐duty trucks, backup power, and distributed generation, cost reduction must be accompanied by longer stack life, lower H_2_ delivery cost, and reliable refueling infrastructure. Fuel‐cell reconversion is therefore more suitable for high‐utilization transport and backup applications than for low‐utilization systems where capital recovery is difficult [72, 78].

SOFCs operate at high temperatures and can achieve high electrical efficiency, especially when waste heat is used in combined heat and power systems. They can use H_2_, syngas, and certain H_2_ carriers after suitable processing, making them relevant for stationary energy systems and industrial sites. However, SOFCs are less suitable for rapid cycling because the high operating temperature can lead to thermal stress, seal and electrode degradation, and longer start‐up times. Their best fit is therefore stationary reconversion, where high efficiency and heat integration are more important than rapid response [79].

For H_2_‐LDES, the choice between PEMFCs, SOFCs, turbines, and engines should be based on discharge duration, start‐up time, ramp rate, efficiency, H_2_ purity, capital cost, and maintenance interval. Fuel cells are suitable where low local emissions, modularity, and part‐load efficiency are required. Turbines may be more appropriate for high‐capacity grid‐scale reconversion, especially where existing gas infrastructure can be adapted. A mixed reconversion portfolio may therefore be required in large H_2_ systems [80].

### 5.2 Hydrogen Combustion in Turbines and Engines

H_2_ combustion in gas turbines and internal combustion engines provides a dispatchable reconversion route for H_2_‐LDES. Gas turbines are relevant for grid‐scale applications because they can deliver high power output, provide reserve capacity, and leverage existing natural gas infrastructure with suitable modifications. H_2_ blending in natural gas turbines can reduce direct CO_2_ emissions, but higher H_2_ fractions require changes to combustor design, fuel delivery, control strategies, and safety systems [81].

The main shortcomings of H_2_ combustion stem from its combustion properties. H_2_ has a high flame speed, wide flammability range, low ignition energy, and high adiabatic flame temperature. These properties increase the risk of flashback, autoignition, thermoacoustic instability, and nitrogen oxide formation. NOx control may require dry low‐NOx combustors, exhaust after‐treatment, or dilution with steam, nitrogen, or water. However, these control strategies can reduce plant efficiency, increase system complexity, and raise operating cost [79, 82].

Material durability is another concern in H_2_‐capable turbines. High‐temperature combustion, frequent start‐stop operation, and rapid load cycling can increase thermal fatigue in combustor liners, transition pieces, turbine blades, and seals. H_2_ can also contribute to embrittlement risk in selected metallic components, especially in fuel delivery and storage systems. Therefore, turbine lifetime cannot be expressed as a single value for all H_2_ applications. It depends on H_2_ fraction, combustor architecture, turbine inlet temperature, cycling frequency, material selection, cooling strategy, and maintenance practice [82].

For LDES applications, the life curve of a H_2_‐capable turbine should be treated as a degradation relationship rather than a fixed lifetime number. As H_2_ fraction and cyclic operation increase, maintenance intervals may shorten unless combustor design, materials, cooling, and NOx‐control systems are adapted. This relationship should be evaluated using operating hours, start‐stop cycles, equivalent operating hours, inspection interval, NOx‐control method, and component degradation rate. H_2_‐capable gas turbines may experience shorter maintenance intervals as the H_2_ fraction and cyclic operation increase, due to higher flame speed, NOx control requirements, flashback risk, and thermal fatigue [83].

**FIGURE 3:**
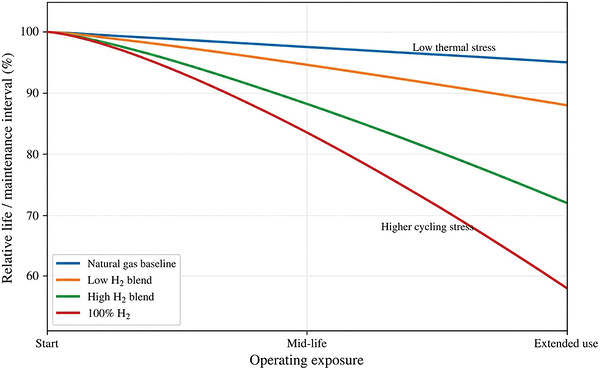
Conceptual life‐degradation framework for H_2_‐capable gas turbines under increasing H_2_ fraction and cyclic operation.

Figure 3 depicts a framework for the life degradation of H_2_‐capable gas turbines related to H_2_ fraction and cyclic operation. The curve illustrates a projected decline in relative life or maintenance intervals as the H_2_ fraction and operational exposure increase. The framework is qualitative, as combustor design, material choices, cooling techniques, NO_x_‐control strategies, and operational duty cycles influence turbine lifespan. Key degradation factors include flashback, thermoacoustic instability, thermal fatigue, NO_x_‐control penalties, and the H_2_ compatibility of fuel‐system components [82, 83].

### 5.3 Industrial Heat and Sector Coupling Applications

H_2_ can support sector coupling by linking power generation, industrial heat, transport, and chemical production. In high‐temperature industries such as steel, refining, and chemical manufacturing, H_2_ can partly replace fossil fuels where direct electrification is technically difficult or costly. However, its practical value depends on the cost of delivered H_2_, the availability of infrastructure, combustion control, safety requirements, and the carbon intensity of H_2_ production [84]. The ability to deploy H_2_ across diverse sectors significantly enhances the value proposition of H_2_‐LDES solutions. By facilitating energy transfer across sectors, H_2_ systems mitigate curtailment of renewable electricity and enhance asset utilization [85].

### 5.4 Efficiency Considerations and System Implications

Despite its multifunctionality, H_2_‐based energy storage exhibits comparatively low round‐trip efficiency compared with battery‐based systems. When losses incurred during electrolysis, compression, storage, and reconversion are considered, the overall round‐trip efficiency generally ranges from 30% to 45% [86]. This efficiency drawback underscores the need to implement H_2_ storage in scenarios where long‐duration storage, seasonal balancing, or sector coupling yields system‐level advantages that outweigh the associated efficiency losses [87].

At the system level, H_2_ reconversion should be used where high energy capacity, extended discharge duration, and grid resilience provide greater value than maximum round‐trip efficiency. Dispatch planning should include electrolysis timing, storage inventory, reconversion availability, demand forecasting, and market signals. This approach can reduce unnecessary cycling and improve the economic use of H_2_ storage assets [88].

## 6 Techno‐Economic and Environmental Assessment

The techno‐economic and environmental performance of H_2_‐LDES depends on the entire production, storage, transport, and reconversion chain. Each stage adds cost, efficiency loss, infrastructure requirement, and environmental burden. A useful assessment must therefore include electricity price, electrolyzer CAPEX, capacity factor, storage cost, reconversion efficiency, life‐cycle emissions, and the system value of long‐duration reliability [89, 90].

**FIGURE 4:**
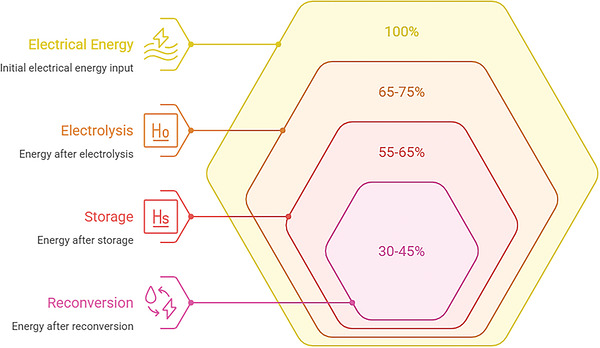
Energy flow and round‐trip efficiency of H_2_‐based long‐duration energy storage systems.

Figure 4 summarizes the main energy losses in the H_2_‐LDES chain. An electrical input of 100% is reduced to about 65%–75% after electrolysis, 55%–65% after storage, and 30%–45% after reconversion through fuel cells or turbines. These losses show why H_2_ is less suitable for short‐duration cycling but remains relevant where long storage duration, large energy capacity, or seasonal balancing is required [86, 90].

### 6.1 Techno‐Economic Performance

#### 6.1.1 Cost Structure of H_2_ Energy Systems

Electricity costs, electrolyzer CAPEX, system utilization rates, and storage infrastructure requirements primarily determine the economic feasibility of H_2_‐based energy systems. Among these determinants, electricity price is the principal cost factor, accounting for approximately 60%–70% of total H_2_ production costs. Given that electrolysis is highly energy‐intensive, access to economically viable renewable electricity is crucial to achieving competitive H_2_ prices [91].

CAPEX for electrolyzers is the second most critical factor affecting economic feasibility. Alkaline electrolyzers typically have a lower initial capital outlay but exhibit diminished operational flexibility, whereas PEM electrolyzers offer rapid response capabilities at a higher cost [92]. Components that constitute the balance of plant, such as power electronics, compression systems, and thermal management, further increase the total system expenditure. The utilization rate of electrolyzers has a substantial impact on economic performance, as reduced operating hours increase levelized H_2_ costs due to underutilized capital investments [93].

Infrastructure for storage and reconversion also contributes to system expenditures, particularly in long‐duration applications. At the same time, compressed and liquefied H_2_ storage systems require considerable upfront capital, and underground storage offers lower operating costs, albeit with geographical limitations. Reconversion technologies, such as fuel cells or turbines, impose additional capital and efficiency burdens that must be accounted for in system‐level economic assessments [94].

### 6.2 Cost Trends and Future Outlook

H_2_ production costs may decline as renewable electricity prices fall, electrolyzer manufacturing scales up, and stack durability improves. However, cost reduction is not determined solely by electrolyzer CAPEX. Storage infrastructure, compression or liquefaction energy, reconversion units, financing conditions, and utilization rate also affect the delivered cost of stored H_2_. For LDES, H_2_ becomes more competitive when the required storage duration exceeds the economic range of short‐duration batteries [95, 96].

Table 5 delineates essential techno‐economic metrics for H_2_‐based extended‐duration energy storage systems, including H_2_ production expenditure, electrolyzer capital investment, round‐trip efficiency, storage expenditure, and system longevity. The aggregated parameters furnish insights into the economic viability and cost determinants influencing large‐scale H_2_ implementation.

**TABLE 5:** Key techno‐economic indicators for H_2_‐based long‐duration energy storage systems.

Parameter	Typical range	Key influencing factors	Remarks	Refs.
H_2_ production cost	2–6 USD kg^−1^	Electricity cost, electrolyzer CAPEX	Expected to drop to 1.5–2 USD kg^−1^	[97]
Electrolyzer CAPEX	400–1200 USD/kW	Technology type, scale	PEM > AEL	[98]
Round‐trip efficiency	30–45%	Storage type, reconversion method	Lower than batteries	[99]
Storage cost	1–15 USD kWh^−1^	Storage medium	Lowest for geological	[100]
System lifetime	15–30 years	Operating conditions	Critical for LCOH	[101]
Operation and maintenance cost	2–5% of CAPEX	Stack degradation	Affects economics	[102]

### 6.3 Life‐Cycle Environmental Impacts

#### 6.3.1 Emissions Associated with H_2_ Production

The environmental performance of H_2_‐based systems depends on both the H_2_ production process and the electricity source used to produce it. Renewable energy‐based H_2_ production through electrolysis will have a wide range of life‐cycle greenhouse gas (GHG) emissions (approximately 1 to 3 kg CO_2_‐equivalent per kilogram of H_2_), whereas H_2_ produced through non‐renewable fossil fuels with no carbon capture technology will likely generate greater than 10 kg CO_2_‐equivalent per kilogram of H_2_; therefore, it would be less environmentally friendly [103, 104].

Although the use of CCS technologies can help to mitigate GHG emissions associated with the H_2_ production process, there are still other GHG emissions that occur during H_2_ production (residual emissions) and methane leaks along the supply chain for H_2_, which may detract from the overall performance metrics of H_2_ as a fuel [105]. Therefore, the climate benefits of using H_2_ as a fuel will depend largely on the degree of decarbonization in the electricity generation mix and the effectiveness of the implemented emission‐reduction strategies [106].

#### 6.3.2 System Boundary and Sustainability Considerations

LCAs of H_2_ systems demonstrate heightened sensitivity to defined system boundaries, equipment operational lifetimes, and underlying assumptions about electricity generation. Beyond emissions generated during operation, it is imperative to account for the environmental impacts associated with electrolyzer manufacturing, material extraction, compression or liquefaction, and end‐of‐life disposal. Investigations that disregard these elements may significantly underestimate the true environmental footprint of H_2_‐based energy systems [107].

From a comprehensive system‐oriented perspective, H_2_‐LDES offers indirect environmental benefits by enabling greater integration of renewable energy sources, mitigating curtailment, and replacing fossil‐fuel‐dependent peaking power plants. When effectively integrated, H_2_ systems not only contribute to emission reductions but also enhance the resilience and sustainability of the broader energy system [108].

Table 6 summarizes the life‐cycle environmental implications of predominant H_2_ production pathways, highlighting GHG emissions across diverse energy sources and system boundaries. The comparison illustrates the environmental benefits of renewable‐based H_2_ relative to fossil‐derived pathways and underscores the significance of the electricity mix and LCA considerations.

**TABLE 6:** Lifecycle GHG emissions of H_2_ production pathways.

H_2_ pathway	Energy source	CO_2_ emissions (kg CO_2_ kg^−1^ H_2_)	System boundary	Environmental impact	Refs.
Green H_2_	Wind/solar	1–3	Cradle‐to‐gate	Very low	[109]
Blue H_2_	Natural gas + CCS	2–6	Well‐to‐gate	Moderate	[110]
Grey H_2_	Natural gas	9–12	Well‐to‐gate	High	[111]
Biomass H_2_	Biomass gasification	0–2	Life‐cycle	Low/negative	[112]
Grid‐powered H_2_	Fossil grid mix	>10	Life‐cycle	Unsustainable	[113]

## 7 System Integration, Reliability, and Digital Optimization

H_2_‐LDES can improve the reliability and flexibility of low‐carbon power systems by shifting energy across daily, weekly, and seasonal timescales. Unlike short‐duration storage, H_2_ can separate the timing of electricity generation, storage, and final use over long periods. Effective integration requires coordinated operation of electrolyzers, storage assets, reconversion units, reliability assessment tools, and digital control systems [114].

**FIGURE 5:**
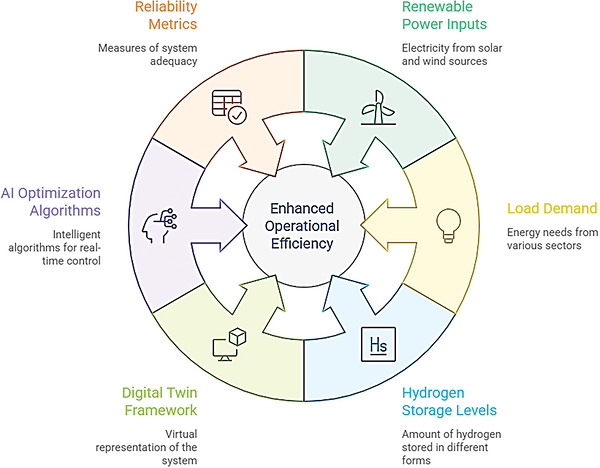
Digital optimization framework for H_2_‐based energy systems integrating renewable inputs, H_2_ storage, and reliability assessment.

Figure 5 illustrates how renewable electricity, H_2_ storage, load demand, and reliability metrics can be connected through digital optimization. LOLE, EENS, and ELCC are used to evaluate resource adequacy, while predictive control can support electrolyzer dispatch, storage scheduling, and reconversion timing under variable renewable generation [115].

### 7.1 Role of H_2_ in Power System Integration

H_2_‐based energy storage systems offer the unique ability to decouple electricity generation from consumption over extended temporal scales. By transforming surplus renewable electricity into H_2_ during periods of excess generation and subsequently reconverting it during periods of deficit, H_2_ systems facilitate a seasonal energy equilibrium that is unattainable with traditional battery storage solutions. This functionality is particularly advantageous in power systems with high wind and solar penetration, where extended periods of low generation can jeopardize system adequacy [116].

Beyond energy shifting, H_2_ systems bolster grid stability by managing peak loads, providing reserves, and supplying backup power. H_2_‐fueled turbines and fuel cells can be deployed during peak‐demand periods or emergencies, thereby reducing dependence on fossil‐fuel peaking facilities. Moreover, H_2_ facilitates sector coupling by linking the electricity, heat, and transport sectors, thereby enhancing overall system flexibility and optimizing asset utilization across the energy value chain [117]. At the system level, H_2_ storage can reduce renewable curtailment, improve the use of generation assets, and support geographically distributed renewable resources when storage, transport, and reconversion infrastructure are available [118].

### 7.2 Reliability Metrics for H_2_‐Based Energy Systems

To measure how H_2_‐based energy storage systems can enhance power system reliability, existing reliability metrics should be used to assess performance. LOLE, EENS, and ELCC are common metrics used to estimate a system's reliability probabilistically and to compare different storage and generation systems [119].

LOLE denotes the anticipated number of hours or days annually during which electricity demand cannot be satisfied, whereas EENS measures the extent of unmet energy demand. H_2_‐based LDES systems have the potential to substantially decrease both metrics by offering dispatchable energy during prolonged supply shortages. ELCC, which quantifies the additional load volume that can be sustained while maintaining a constant level of reliability, is particularly beneficial for assessing the firm capacity contribution of H_2_ storage [120].

In contrast to batteries, whose ELCC declines rapidly with increasing storage duration, H_2_ systems retain a relatively stable capacity over prolonged periods due to their ability to store substantial energy. This characteristic renders H_2_ particularly advantageous for seasonal reliability and long‐term resource adequacy planning. However, the precise estimation of these metrics necessitates comprehensive modeling of H_2_ production constraints, storage availability, reconversion efficiency, and operational strategies [121].

### 7.3 Digital Optimization and Intelligent Regulation of H_2_ Systems

The escalating complexity of H_2_‐based energy infrastructures has accelerated the integration of digital technologies to enhance operational efficiency and reliability. Digital twins, AI, and sophisticated control algorithms are progressively being employed to model, monitor, and optimize H_2_ production, storage, and utilization in real‐time environments [122].

Digital twins facilitate the development of virtual representations of tangible H_2_ systems, thereby enabling operators to simulate performance metrics, forecast potential failures, and optimize operational parameters in response to fluctuating demand and renewable energy generation conditions [123]. AI methodologies, including machine learning (ML) and reinforcement learning, are used to enhance electrolyzer dispatch, manage storage effectively, and schedule reconversion processes, thereby elevating system efficiency and mitigating operational expenditures [124].

Predictive maintenance algorithms significantly enhance system reliability by detecting component deterioration and scheduling maintenance activities before failures occur. When integrated with energy management systems and market indicators, digital optimization tools enable H_2_ systems to participate dynamically in electricity markets, ancillary services, and capacity mechanisms [125].

Digital tools can improve H_2_‐LDES operation when they are connected to measurable outcomes such as lower curtailment, reduced degradation, improved reliability, or lower operating cost. Their value should therefore be assessed using operating data, dispatch performance, maintenance records, and reliability metrics rather than general claims of optimization [126].

## 8 Challenges and Future Research Directions

H_2_‐LDES faces technical, economic, infrastructural, and regulatory barriers that differ from those of short‐duration storage. The main challenges are not limited to H_2_ production. They include electrolyzer degradation under variable operating conditions, energy losses during compression or liquefaction, storage‐site limitations, reconversion efficiency, H_2_ purity requirements, safety management, high capital costs, and the absence of market mechanisms that reward multi‐day or seasonal reliability. These barriers need to be assessed across the full chain of production, storage, transport, reconversion, and end use [120, 127].

Recent institutional and system‐planning assessments indicate that H_2_ storage deployment is constrained by cost, infrastructure readiness, project utilization, storage‐site validation, and demand creation. These barriers show that H_2_‐LDES development requires more than component‐level improvement. It also requires coordinated planning of renewable electricity supply, electrolyzer operation, H_2_ hubs, geological storage, reconversion assets, certification systems, and long‐term market incentives [127].

### 8.1 Challenges in H_2_‐Based Long‐Duration Energy Storage

#### 8.1.1 Technical and Operational Challenges

The main technical challenge in H_2_‐LDES is maintaining efficiency and durability across a multi‐stage energy chain. Electrolyzers coupled with variable renewable electricity may experience frequent ramping, low‐load operation, start‐stop cycling, and pressure fluctuations. These operating conditions can increase membrane degradation, catalyst deactivation, gas crossover, and balance‐of‐plant stress. The issue is more severe in systems that rely on intermittent electricity than in systems supplied by stable industrial power [128].

Round‐trip efficiency remains a major limitation. Losses occur during electrolysis, compression or liquefaction, storage, carrier conversion where applicable, and reconversion through fuel cells, turbines, or engines. As a result, the overall round‐trip efficiency of H_2_ storage is generally lower than that of battery storage and is often reported to be 30–45%. This does not exclude H_2_ from energy storage. Still, it means that H_2_‐LDES should be used where storage duration, energy capacity, seasonal balancing, or sector coupling provides system value that cannot be supplied economically by short‐duration storage [129].

#### 8.1.2 Infrastructure and Scalability Constraints

Infrastructure is a central constraint for H_2_‐LDES deployment. Large systems require electrolyzers, water supply, power electronics, compressors, storage facilities, pipelines or transport systems, reconversion units, safety systems, and monitoring infrastructure. Geological storage can reduce the cost of large energy capacity, but it is geographically constrained and requires site‐specific assessment. Salt caverns provide high deliverability where available, while depleted reservoirs and aquifers require additional evaluation of leakage, cushion gas, microbial activity, and geochemical interaction [130].

H_2_ transport and distribution are less developed than electricity and natural gas networks in many regions. Repurposing natural gas pipelines may reduce infrastructure cost in selected cases, but H_2_ embrittlement, leakage, compressor compatibility, metering, safety standards, and blending limits must be evaluated. Where pipelines or geological storage are unavailable, ammonia, LH_2_, or LOHC supply chains may be more practical, although they introduce conversion losses and additional safety requirements [131].

#### 8.1.3 Economic and Policy Barriers

The economic feasibility of H_2_‐LDES depends on electricity prices, electrolyzer utilization, capital costs, storage costs, reconversion costs, financing conditions, and the value assigned to long‐duration reliability. Low electrolyzer utilization can increase H_2_ cost because capital recovery is spread across fewer operating hours. Very high utilization may require grid electricity or firm renewable supply, which can increase cost or emissions depending on the power mix. Cost assessment should therefore include both H_2_ production cost and system‐level storage value [132].

Policy and market design remain incomplete for many H_2_‐LDES applications. Current electricity markets often compensate energy, capacity, or ancillary services separately, but they may not adequately value multi‐day adequacy, seasonal balancing, resilience, or avoided renewable curtailment. H_2_ certification, carbon accounting, safety regulation, storage permitting, and long‐term offtake agreements are also needed to reduce investment risk. Without these mechanisms, technically feasible H_2_ storage projects may remain difficult to finance [133].

### 8.2 Future Research Priorities for H_2_‐LDES

Future research should focus on improving the durability, efficiency, and cost of electrolyzers under realistic renewable‐coupled operation. Laboratory performance should be complemented by long‐duration testing under variable load, start‐stop cycles, pressure fluctuation, and partial‐load operation. Research is also needed on low‐cost catalysts, stable membranes, stack diagnostics, and balance‐of‐plant designs that reduce degradation during dynamic operation. These improvements are necessary because electrolyzer lifetime and replacement costs strongly affect the levelized cost of H_2_ [134].

Storage research should move beyond material‐level capacity and address system‐level performance. Geological H_2_ storage requires a better understanding of H_2_ loss, microbial conversion, cushion gas demand, withdrawal rates, leakage monitoring, and long‐term integrity. LOHC and ammonia storage require improved catalysts, lower deH_2_ation energy, safer handling methods, and better integration with reconversion systems. Future studies should compare these storage options using common boundaries that account for conversion losses, storage duration, infrastructure costs, and safety requirements [135].

Digitalization can support H_2_‐LDES operation, but it should be linked to measurable system outcomes. Digital twins, predictive control, and machine‐learning models should be used to improve electrolyzer dispatch, storage scheduling, reconversion timing, fault detection, and maintenance planning. These tools need validation using real operating data from H_2_ hubs, pilot plants, and integrated renewable‐H_2_ systems. Future work should report whether digital control reduces cost, degradation, curtailment, or reliability risk rather than treating digitalization as a general benefit [136].

System‐planning studies should include reliability metrics such as LOLE, EENS, and ELCC when evaluating H_2_‐LDES. These metrics can show whether H_2_ storage contributes firm capacity during extended renewable shortfalls. Future models should include electrolyzer constraints, storage inventory, reconversion efficiency, fuel‐cell or turbine availability, renewable variability, and market participation. This approach will enable comparison of H_2_ storage with batteries, pumped hydro, demand response, transmission expansion, and other flexibility options using consistent adequacy criteria [137].

Demonstration projects are needed to connect component‐level performance with system‐level operation. Priority should be given to projects that integrate renewable electricity, electrolysis, large‐scale storage, reconversion, and end‐use demand into a single operating system. Such demonstrations should report capacity factor, H_2_ loss, storage cycling, reconversion efficiency, downtime, maintenance cost, safety events, and delivered energy cost. Transparent reporting will improve comparison across projects and reduce uncertainty in investment decisions [138].

Policy research should address market design, certification, safety, and infrastructure planning. H_2_‐LDES requires long‐term revenue streams because many benefits occur during rare but important reliability events. Capacity markets, contracts for difference, H_2_ certification, carbon pricing, storage incentives, and public support for shared infrastructure may all be needed depending on the region. Future policy frameworks should distinguish H_2_ for short‐duration backup, industrial decarbonization, transport, and seasonal power‐system storage because these applications have different cost structures and public benefits [139].

Table 7 summarizes the main barriers, evidence gaps, and research priorities for H_2_‐LDES. The table emphasizes that deployment barriers span production, storage, efficiency, infrastructure, digitalization, and policy. Addressing these issues requires consistent field data, common reporting boundaries, and market mechanisms that recognize multi‐day and seasonal reliability services.

**TABLE 7:** Technical barriers, evidence gaps, and research priorities for H_2_‐based long‐duration energy storage.

Category	Current barrier	Evidence gap	Research priority	Refs.
Electrolysis	High CAPEX and degradation under variable load	Limited long‐duration field data under renewable‐coupled operation	Dynamic durability testing, lower‐cost catalysts, and stack diagnostics	[140]
Storage	Energy loss, safety risk, and site dependence	Limited comparable data for salt caverns, reservoirs, ammonia, and LOHC systems	Common storage assessment criteria covering duration, cost, loss, and safety	[141]
Efficiency	30%–45% round‐trip efficiency in many H_2_ storage chains	Inconsistent system boundaries across studies	Standardized efficiency reporting from electricity input to delivered output	[142]
Infrastructure	Limited pipelines, refueling systems, and geological storage access	Unclear regional infrastructure readiness	H_2_ hubs, shared pipelines, storage mapping, and port‐linked carrier chains	[143]
Digitalization	Limited validation of AI and digital twins with operating data	Insufficient evidence of cost, reliability, or degradation reduction	Pilot‐scale validation of predictive control and maintenance models	[144]
Policy and markets	Weak revenue mechanisms for multi‐day and seasonal reliability	Limited valuation of resilience and avoided curtailment	Capacity‐market design, certification, carbon accounting, and long‐term offtake	[145]

## 9 Conclusions

This review examined H_2_‐based LDES across production, storage, carrier conversion, reconversion, techno‐economic assessment, LCA, reliability contribution, and digital optimization. The analysis shows that H_2_ is most relevant when storage duration extends beyond daily balancing and when large energy capacity, low time‐dependent losses, seasonal shifting, or sector coupling is required. For short‐duration services, batteries and other electrochemical systems are often more efficient. For multi‐day, weekly, and seasonal storage, H_2_ can provide value when the full system is designed around realistic efficiency, cost, infrastructure, and reliability constraints.

Electrolysis is the main low‐carbon production pathway when powered by renewable or other low‐carbon electricity. Its suitability depends on capital cost, electricity price, capacity factor, stack lifetime, and degradation under dynamic operation. Thermochemical and hybrid routes may support regional deployment where feedstock, carbon capture, waste heat, or biomass resources are available. Still, their climate benefit depends on life‐cycle emissions and carbon‐accounting boundaries.

Storage selection is strongly region dependent. Compressed H_2_ is more suitable for decentralized and short‐ to medium‐duration applications, while LH_2_, LOHCs, and ammonia are more relevant for transportable storage and international supply chains. Geological storage offers the strongest technical fit for large, stationary, and seasonal H_2_‐LDES when suitable salt caverns, depleted reservoirs, or aquifers are available. Storage planning should therefore be based on regional geology, infrastructure, safety requirements, and end‐use demand rather than a single general ranking.

Fuel cells, turbines, and engines provide different reconversion options. Fuel cells are suitable for distributed power, backup systems, and selected electric mobility applications where rapid response and low local emissions are required. H_2_ turbines are better suited to high‐capacity grid‐scale dispatch but pose challenges related to NO_x_ control, flashback, thermoacoustic instability, material durability, and maintenance. These limitations indicate that reconversion technology should be selected based on duty cycle, discharge duration, ramping requirements, and fuel quality.

The main barriers to H_2_‐LDES are high cost, low round‐trip efficiency, infrastructure gaps, storage‐site limitations, safety management, and incomplete market mechanisms for long‐duration reliability. Future research should prioritize field validation, standardized techno‐economic and life‐cycle boundaries, geological storage assessment, durable electrolyzers, lower‐cost fuel cells, H_2_‐compatible turbines, and data‐based digital control. With region‐specific design and suitable market support, H_2_‐LDES can contribute to high‐renewable power systems where long storage duration and large energy capacity are more important than maximum round‐trip efficiency.

## Funding

The authors extend their appreciation to the Deanship of Research and Graduate Studies at King Khalid University for funding this work through the Large Research Project under grant number RGP2/526/46.

## Conflicts of Interest

The authors declare no conflicts of interest.

## Data Availability

The data that support the findings of this study are available on request from the corresponding author. The data are not publicly available due to privacy or ethical restrictions.
